# Lycopene improves on basic hematological and immunological parameters in diabetes mellitus

**DOI:** 10.1186/s13104-019-4841-8

**Published:** 2019-12-12

**Authors:** Ejike Daniel Eze, Adam Moyosore Afodun, Josephine Kasolo, Keneth Iceland Kasozi

**Affiliations:** 1 Department of Biomedical Sciences, Kabale University, Kabale, Uganda; 2 Department of Physiology, Faculty of Biomedical Sciences, Kampala International University Western Campus, Box 71, Bushenyi, Uganda; 3 Department of Anatomy, Faculty of Biomedical Sciences, Kampala International University Western Campus, Box 71, Bushenyi, Uganda; 4 Department of Physiology, College of Health Sciences, Makerere University, Box 7062, Kampala, Uganda

**Keywords:** Antioxidants, Diabetes, Anemia, Carotenoids, Blood

## Abstract

**Objective:**

Diabetes is associated with an upset of hematological and immunological parameters in humans, however information on the effects of Lycopene is scarce. The aim of the study was to gain information on basic changes in hematological parameters as markers for safety since anemia as a complication in diabetic chemotherapy has been reported.

**Results:**

Lycopene had anti-anemic effects and improved on the immune status of diabetic rats and these observations were dose independent. There was a decrease in neutrophil, low neutrophil–lymphocyte ratio and platelet counts and stable albumin, globulin levels. Lycopene could exert its protective effects through a balance of basic hematological physiological variables.

## Introduction

Anemia has been identified as a major complication of diabetes mellitus (DM) in humans [1] and vitamin supplementation using folic acid and vitamin B12 has been associated with improved patient outcomes, although a lot remains to be established in this field [2]. In blood, a significant reduction in vitamin B12 (cobalamin) due to its malabsorption in Type 1 Diabetes Mellitus (T1DM) would be associated with anemia since this is a maturation factor needed during erythropoiesis [3]. Metformin use has also been associated with low vitamin B12 levels [4] demonstrating a need for novel therapies to manage complications in DM therapy. Lycopene is a phytochemical carotenoid, red in color, lipophilic and naturally occurring in many natural products especially in tomatoes [5]. Carotenoids have been associated with strong antioxidant activity and this is responsible for their therapeutical benefits in humans following a heavy vegetable intake [6]. Previously, the hypoglycemic and pancreatic protective effects of Lycopene have been established [7], however information on its effects on hematological parameters in anemia is scarce. Hematological indices i.e. mean corpuscular hemoglobin concentration (MCVH) has been shown to be higher in diabetic patients and the mean corpuscular hemoglobin concentration negatively correlated with blood pressure demonstrating the importance of hematological indices in diabetes [8]. In severe diabetic human patients, anemia has been associated with low hemoglobin (Hb) levels [9] demonstrating its importance as a clinical marker in this regard. Since glycated hemoglobin (A1C) is a reference for long-term glucose monitoring, its association with DM complications leads to its ability to interfere with Hb metabolism, the glycated albumin reflects short-term glycemia and is not influenced by situations that falsely alter A1C levels [10] however, information on Lycopene induced albumin changes (due to alterations in Hb metabolism) is scarce. Serum albumin can be estimated using the bromocresol green (BCG) and bromocresol purple (BCP) methods and these methods may lead to serious confusions especially at low albumin levels and an overestimation has been associated with serum acute phase proteins [11]. High globulin levels have also been reported as a risk factor to diabetes [12] and these would be associated with beta globulins while gamma globulins are decreased in diabetes, however the scarcity of information on the role of Lycopene on blood parameters created a rationale for the study. Lycopene would alter basic hematological and immunological parameters and this formed the objective for the current study.

## Main text

### Methods

#### Study design

Male Wistar rats (N = 30) weighing 150–200 g were kept as previously described with 24 h of day light [13] at Kampala International University Western Campus. Diabetes mellitus was induced by using 60 mg/kg of Streptozotocin (Sigma^®^) in olive oil and after confirmation of hypoglycemia [13] and insulin levels (Additional file 1: Figure S1), rats were divided randomly using MS Excel version 2013 by assigning a random number from 1–5 into experimental groups as previously described [14] i.e. 5 rats per experimental group. Thirty rats were divided into a negative control (n = 5) which was administered with olive oil, and diabetic rats were used in positive control (n = 5) with olive oil at 0.5 ml/kg, the comparative control (n = 5) administered with glibenclamide at 2 mg/kg and three experimental groups of Lycopene (Pittsburgh, USA) i.e. low (n = 5), medium (n = 5) and high dose (n = 5) of lycopene at 10 mg/kg, 20 mg/kg and 40 mg/kg of Lycopene per os for 28 days. Lycopene was reconstituted in olive oil to appropriate working dosage as previously described [15]. The animals were kept in standard plastic cages, provided water and feeds adlibitum and animal were assessed on hematological parameters in line with ARRIVE guidelines.

#### Blood sample collection and serum preparation

After the last day of treatment all animals were euthanized using sodium pentobarbitone injected intraperitoneally [13] and 5 ml of blood was collected through cardiac puncture into heparinized sample bottles for hematological analysis. The other portion of blood sample collected into specimen bottles and allowed to clot, and separated by centrifugation at 2000*g* for 10 min using Centrifuge Hettich (Universal 32, Made in Germany). The supernatant obtained was used for the determination of erythrocyte osmotic fragility test.

#### Determination of serum total protein, albumin, globulin and albumin-globulin ratio

The serum total protein was determined using a Eurochem^®^ total protein test kit according to the manufacturer’s recommendations [16]. Serum albumin which quantitatively binds bromocresol green (BCG) to form an albumin-BCG complex was measured as an endpoint reaction at 596 nm [11]. Globulin was obtained by subtracting the albumin from the total protein and the albumin-globulin ratio was obtained subsequently.

#### Determination of hematological parameters

These were determined using an automated analyzer using the manufacturer’s manual [17]. Parameters of interest were red blood cells (RBC) counts, white blood cells (WBC) counts, haemoglobin (Hb) concentration, packed cell volume (PCV), mean corpuscular volume (MCV), mean corpuscular hemoglobin (MCH) and mean corpuscular hemoglobin concentration (MCHC).

#### Data analysis

Data was tested for normalcy and parametric tests were conducted using Graph Pad Prism Version 6 and information was presented as figures and a Table. ANOVA test was done and Tukey’s test was used to determine sources of variation and significant differences (P < 0.05) were indicated with different superscripts i.e. letters a and b.

### Results

#### Lycopene improves on hematolgoical indices during diabetes mellitus

**Fig. 1:**
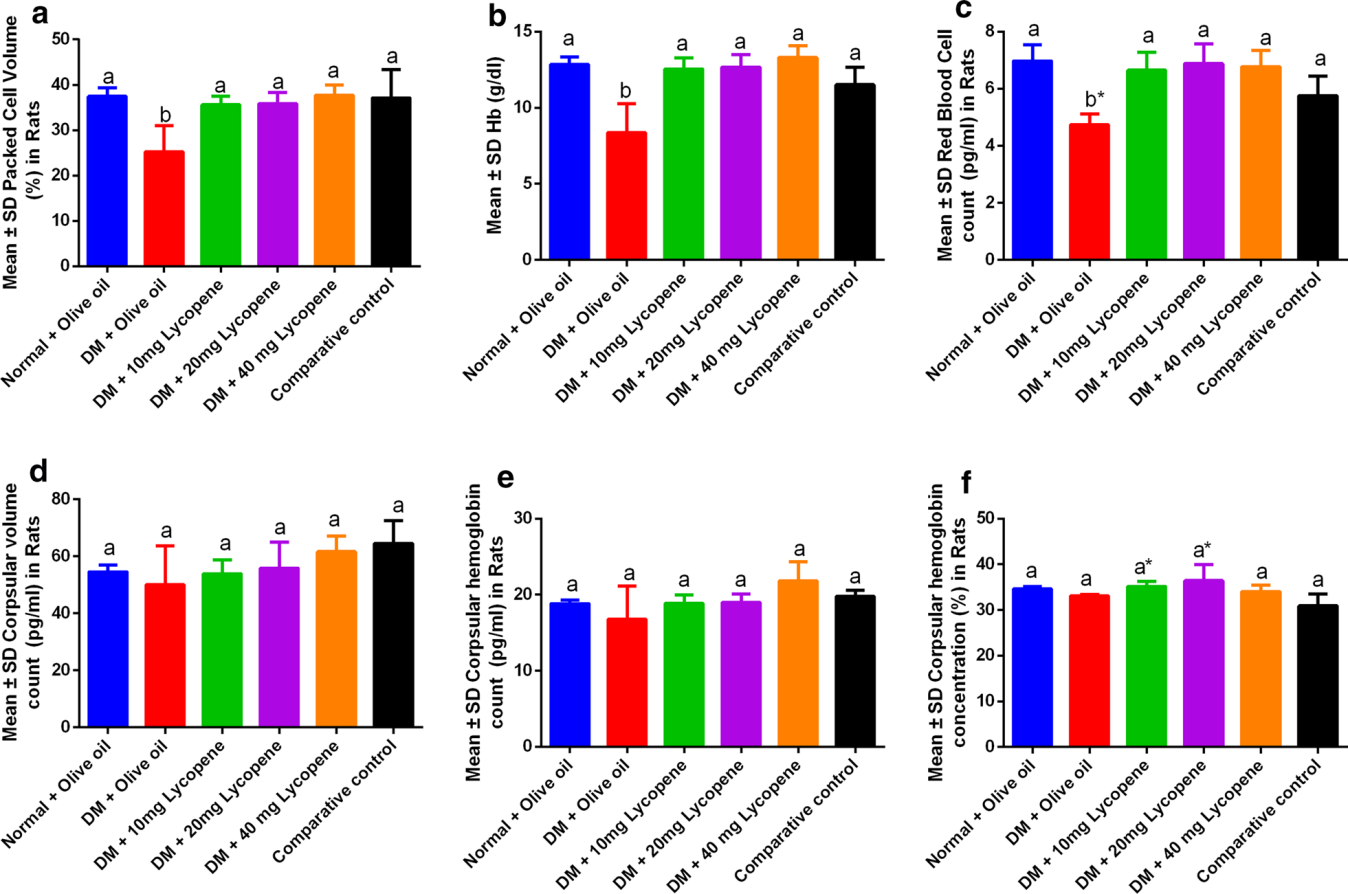
Changes in hematological indices following Lycopene administration in male Wistar rats. **a** Packed cell volume (PCV), **b** Hb concentration, **C** Red blood cell count (RBCs), **d** Mean corpuscular volume (MCV), **e** Mean corpuscular hemoglobin count (MCHC) and **f** Mean corpuscular hemoglobin concentration (MCHC). Since anemia is defined by a significant decrease in PVC, RBCs, and MCV, these acted as markers for anemia in the experimental animals

Lycopene administration was associated with a significant increase in the packed cell volume, hemoglobin levels, and red blood cell counts (Fig. 1a–c) while no significant differences were seen in the mean corpuscular volume (MCV), mean corpuscular hemoglobin (MCH) and mean corpuscular hemoglobin concentration (MCHC). There were no significant effects in lycopene activity at higher concentrations demonstrating that effects observed are dose independent as shown in Table 1.

**Table 1:** Inferential analysis on hematological parameters following Lycopene administration in diabetic Wistar rats

Tukey’s multiple comparisons test	PCV	Hb	RBC counts	MCV	MCH	MCHC	WBC	LYMP	NEUT	N:L	Platelets	Total protein	Albumin	Globulin
	Adjusted P values													
Normal + olive oil vs. DM + olive oil	0.0006	< 0.0001	< 0.0001	0.9469	0.6628	0.8080	0.4535	0.3125	0.8834	0.0001	0.0032	0.0005	0.9357	0.0202
Normal + olive oil vs. DM + 10 mg Lycopene	0.9737	0.9984	0.9491	> 0.9999	> 0.9999	0.9973	0.8742	> 0.9999	0.0190	0.1455	0.2215	0.8463	0.9099	> 0.9999
Normal + olive oil vs. DM + 20 mg Lycopene	0.9849	0.9999	0.9998	0.9999	> 0.9999	0.7016	> 0.9999	> 0.9999	0.5622	0.6296	0.9999	0.8907	0.9998	0.9825
Normal + olive oil vs. DM + 40 mg Lycopene	> 0.9999	0.9836	0.9931	0.7344	0.3103	0.9968	> 0.9999	> 0.9999	0.5846	0.1947	0.8684	0.8463	0.8423	0.9997
Normal + olive oil vs. comparative control	> 0.9999	0.4128	0.0370	0.3898	0.9827	0.0588	0.5872	0.4467	0.6953	0.9995	0.9693	0.1483	0.0328	0.6926
DM + olive oil vs. DM + 10 mg Lycopene	0.0037	< 0.0001	0.0004	0.9733	0.6364	0.5423	0.0664	0.2702	0.1818	0.0603	< 0.0001	< 0.0001	0.4054	0.0252
DM + olive oil vs. DM + 20 mg Lycopene	0.0030	< 0.0001	< 0.0001	0.8667	0.6098	0.1136	0.3617	0.3958	0.9909	0.0061	0.0057	< 0.0001	0.8423	0.0040
DM + olive oil vs. DM + 40 mg Lycopene	0.0004	< 0.0001	0.0002	0.2446	0.0149	0.9684	0.4115	0.4281	0.9932	0.0427	0.0002	< 0.0001	0.3179	0.0389
DM + olive oil vs. comparative control	0.0008	0.0014	0.1049	0.0832	0.2804	0.5049	0.0209	0.0065	0.9990	0.0003	0.0005	< 0.0001	0.0037	0.3498
DM + 10 mg Lycopene vs. DM + 20 mg Lycopene	> 0.9999	> 0.9999	0.9886	0.9989	> 0.9999	0.9170	0.9319	0.9998	0.4529	0.9129	0.1441	> 0.9999	0.9720	0.9687
DM + 10 mg Lycopene vs. DM + 40 mg Lycopene	0.9522	0.8851	0.9995	0.6552	0.3314	0.9363	0.9024	0.9995	0.4320	> 0.9999	0.8281	> 0.9999	> 0.9999	> 0.9999
DM + 10 mg Lycopene vs. comparative control	0.9887	0.6581	0.2117	0.3208	0.9871	0.0216	0.9946	0.5014	0.3346	0.2556	0.6344	0.7363	0.2431	0.7489
DM + 20 mg Lycopene vs. DM + 40 mg Lycopene	0.9698	0.9418	0.9996	0.8571	0.3532	0.4187	> 0.9999	> 0.9999	> 0.9999	0.9565	0.7497	> 0.9999	0.9357	0.9224
DM + 20 mg Lycopene vs. comparative control	0.9945	0.5506	0.0643	0.5294	0.9906	0.0020	0.6884	0.3575	> 0.9999	0.8122	0.9086	0.6738	0.0569	0.3021
DM + 40 mg Lycopene vs. comparative control	0.9999	0.1374	0.1176	0.9915	0.7016	0.1505	0.6325	0.3283	> 0.9999	0.3288	0.9993	0.7363	0.3179	0.8480

*DM* Diabetes mellitus, *PCV* packed cell volume, *Hb* hemoglobin, *RBC* red blood cell, *LYMP* lymphocytes, *MCH* mean corpuscular haemoglobin, *MCHC* mean corpuscular haemoglobin concentration, *MCV* mean corpuscular volume, *N:L* neutrophil to lymphocyte ratio

#### Lycopene improves on the immune status in diabetic rats

**Fig. 2:**
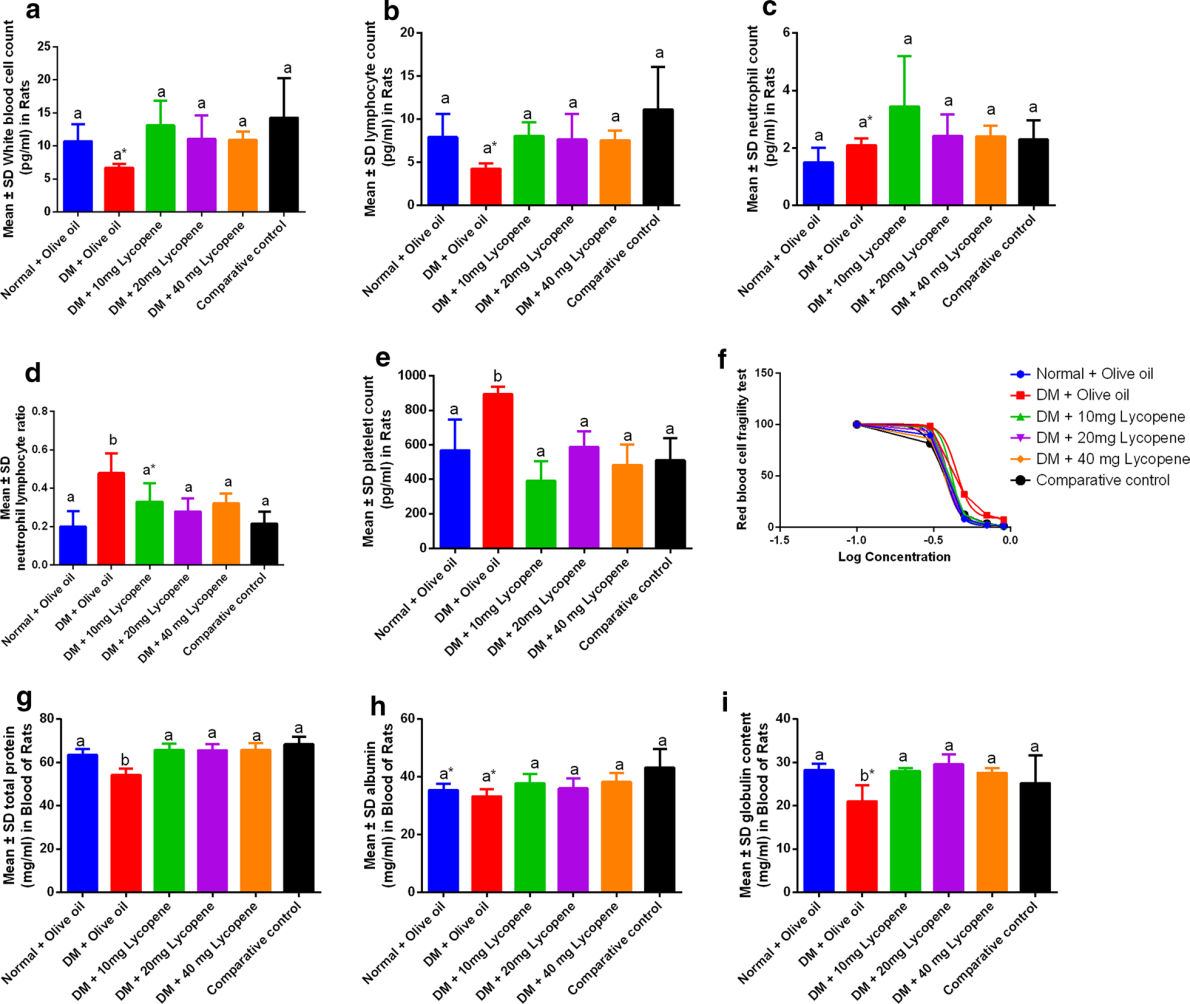
Changes in immunological parameters following Lycopene administration in male Wistar rats. **a** White blood cell counts (WBCs), **b** Lymphocyte count, **c** Neutrophil counts, **d** Neutrophillymphocyte ratio, **e** Platelet count, **f** Red blood cell fragility test, **g** Total protein, **h** blood albumin, **I** globulin content. The immunological activity was reflected by changes in neutrophils, WBCs, and lymphocytes and in diabetes mellitus, these were relatively suppressed. The lymphocyte ratio demonstrated that DM was characterized with significantly elevated neutrophil counts (**d**) and this was associated with an elevated platelet count. A low total protein count (**g**) was due to significantly low globulin levels (**I**) while RBC fragility was lowest in the healthy rats and highest in the diabetic groups

Lycopene administration was associated with an increase in white blood cells and lymphocyte counts (Fig. 2a, b), however, a decrease in neutrophils was observed although no significant differences were observed (Fig. 2c). Lycopene was also associated with significant decrease in neutrophil–lymphocyte ratio and platelet counts (Fig. 2d, e). Furthermore, Lycopene was associated with significant increase in blood total protein and globulin levels (Fig. 2g, i) however, no significant differences were found in the blood albumin levels and fragility tests (Fig. 2h, f).

#### Fragility test non-liner regression

The study showed that the effective concentration which had effects on 50% RBC activity was found to be 0.38, 0.44, 0.41, 0.39, 0.37 units for normal + olive oil, DM + olive oil, 10 mg, 20 mg, 40 mg lycopene and comparative control demonstrating the safety of concentrations used in the study. Significant differences in normal and DM with olive oil against the comparative control for albumin. No significant differences in DM + olive oil and comparative control on globulin levels (P = 0.3498).

### Discussion

The study demonstrated that Lycopene had anti-anemic effects (Fig. 1) demonstrating its importance in the management of anemia associated with diabetes mellitus (Additional file 1: Figure S1). This is important since little is known about the role of carotenoids on hematological indices [1, 2] and in this study, Lycopene could upregulates erythropoiesis which is associated with the maturation factor [3]. In addition, the strong hematoprotective effects of Lycopene (Fig. 1) strengthen its therapeutical effects observed in diabetes and in pancreatic protection [7]. This is important since the conventional drug metformin interferes with vitamin B12 absorption [4], demonstrating its relevance in integrative and alternative medicine. No changes in the mean corpuscular volume, mean corpuscular hemoglobin, mean corpuscular hemoglobin concentration (Table 1), demonstrating the safety of Lycopene at varying concentrations on blood. In diabetes, these hematological indices are often high [8], thus the use of Lycopene in integrative medicine would help lessen side effects and toxicities associated with conventional therapies in diabetes.

In the body, white blood cells play a crucial role in immunity, thus an increase in WBC and lymphocyte counts (Fig. 2a, b) improves on the immune status of diabetic rats and makes them more resilient against opportunistic infections. Since these observations were dose independent in Lycopene, the strong antioxidant activity would be responsible for a favorable immune response as is common with many vegetables and fruits due to their high carotenoid content [6]. A decrease in neutrophil levels showed that no major inflammatory processes were ongoing in diabetic rats thus revalidating the therapeutical potential of Lycopene in diabetes. A significantly low neutrophil–lymphocyte ratio and platelet counts showed that the risk of liver and pancreas damage associated with diabetes was minimal [7]. In diabetes, differences in beta and gamma globulins and low albumin levels have been reported [11, 12] and the ability of Lycopene to maintain these within normal ranges demonstrated its therapeutical advantages in alternative and complimentary medicine.

### Limitations

The study worked on basic markers hematological parameters thus a follow study on iron content, ferritin levels, erythropoietin, secondary messengers, antioxidant status, inflammatory cytokines would offer a clearer insight on the molecular mechanism of Lycopene activity in diabetes. In this study we offer basic information on the use of natural products rich in Lycopene in alternative medicine, however, effects of Lycopene in several body organs remains to be established.

## Supplementary information

**Additional file 1: Figure S1.** Changes in blood glucose and insulin levels during the experimental period.

## Data Availability

Information used in the study can be accessed on https://figshare.com/s/472d34dc4f4de9ac93cc.

## Abbreviations

A1C: glycated hemoglobin
BCG: bromocresol green
BCP: bromocresol purple
DM: diabetes mellitus
Hb: hemoglobin
LYMP: lymphocytes
MCH: mean corpuscular haemoglobin
MCHC: mean corpuscular haemoglobin concentration
MCV: mean corpuscular volume
mg/kg: milligrams per kilogram of body weight
nm: nanometers
PCV: packed cell volume
RBC: red blood cells
WBC: white blood cells
