# Supplementary material for: Lycopene improves on basic hematological and immunological parameters in diabetes mellitus
Source: BMC Res Notes. 2019 Dec 12;12:805. doi: 10.1186/s13104-019-4841-8 (PMC6909647; doi:10.1186/s13104-019-4841-8)
Supplement: Supplementary file 1 — Additional file 1: Figure S1. Changes in blood glucose and insulin levels during the experimental period. [file 13104_2019_4841_MOESM1_ESM.pdf]

Additional 1: Changes in blood glucose and insulin levels during the experimental period

Hyperglycemia was determined on a weekly basis throughout the experimental period and this was recorded using a digital glucometer (Accu-chek Advantage) while insulin levels were determined using a radio-immunoassay using Mercodia Ultrasensitive Rat Insulin ELISA kits (10-1251-01) following the manufacturer's recommendations. Hyperglycemia was confirmed in all experimental animals by week 0 following administration of STZ while insulin levels were significantly suppressed (**S1**).

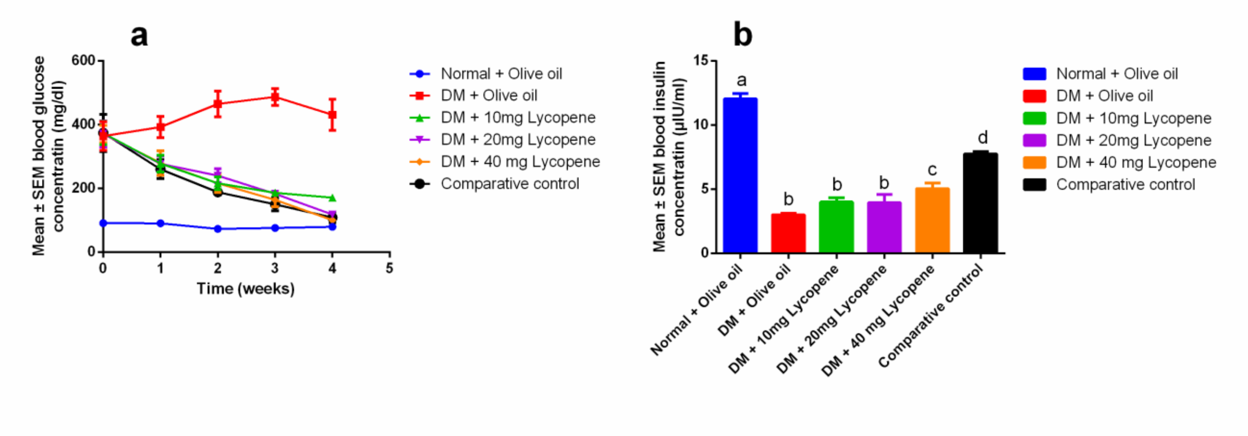

Figure S1. Changes in blood glucose and insulin levels during the experimental period. An increase in blood glucose levels in the diabetic group without treatment (positive control) was observed while in rats having diabetes mellitus and on treatment with Lycopene, blood glucose levels gradually reduced and by the 4th week, levels were comparable to those in rats without diabetes. Furthermore, blood insulin levels were lowest in the diabetic and olive oil group (positive control) and insulin concentrations increased with an increase in Lycopene concentration administered (Figure S1b).
